# Quality of Measurement Properties in Patient Reported Outcomes Used in Adult Liver Transplant Candidates and Recipients: a Systematic Review

**DOI:** 10.3389/ti.2025.14497

**Published:** 2025-10-02

**Authors:** Samira E. M. van Knippenberg, Sarah F. Powell-Brett, Kunal Joshi, Víola B. Weeda, Hermien Hartog

**Affiliations:** ^1^ Department of Surgery, Amsterdam University Medical Centers, Amsterdam, Netherlands; ^2^ The Liver Unit, Queen Elizabeth Hospital Birmingham, Birmingham, United Kingdom; ^3^ Centre for Liver and Gastrointestinal Research, Institute of Immunology and Immunotherapy, University of Birmingham, Birmingham, United Kingdom; ^4^ Department of Surgery, Hôpital Universitaire de Bruxelles, Bruxelles, Belgium; ^5^ Department of Surgery, Section of HPB and Liver Transplantation, University Medical Center Groningen, Groningen, Netherlands

**Keywords:** patient reported outcome measures, liver transplantation, quality of life, measurement properties, surgery

## Abstract

**Objective:**

Patient Reported Outcome Measures (PROMs) are increasingly recognized in liver transplant (LT)-patients, yet recent evaluations of their quality are lacking. This systematic review gives a comprehensive overview of available PROMs in adults awaiting or undergoing LT and their measurement properties.

**Method:**

A systematic search in MEDLINE, EMBASE, PubMed, and COCHRANE (01/2010–08/2023) included studies involving adult LT-candidates and/or recipients utilizing PROMs with original evaluations of measurement properties. The COnsensus-based Standards for the selection of health Measurement INstruments (COSMIN) was used to ascertain the quality of measurement properties.

**Results:**

In total, 23 studies encompassing 35 PROMs were identified, including nine disease-specific and 26 generic PROMs. The *(Short-form) Liver Disease Quality of Life* ((SF-)LDQoL), *Transplant Effects Questionnaire* (TxEQ) and *Post-Liver Transplant Quality of Life* (pLTQ) were the most utilized disease-specific PROMs. Most studies demonstrated low-quality evidence for measurement properties. *pLTQ* demonstrated high-quality evidence for internal consistency, reliability, and responsiveness; the generic *Hospital Anxiety and Depression Scale (HADS)* showed strong evidence for internal consistency and construct validity.

**Conclusion:**

Measurement properties in LT-patients remains of low-quality. *pLTQ* stands out for its superior methodological quality among disease-specific PROMs. For future studies, there is a strong recommendation to focus more on patients’ subjective measures and their measurement properties.

## Graphical Abstract

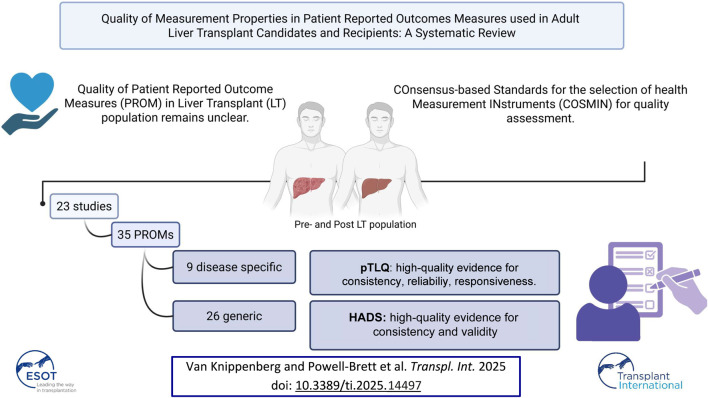

## Introduction

The field of liver transplantation (LT) is rapidly evolving. Over the last 10 years, more than 8,000 liver transplants have been performed in the United Kingdom with excellent long-term outcomes. In the United Kingdom, elective transplant procedures exhibit respective one- and 5-year survival rates of 94% and 81%, while urgent transplant cases demonstrate corresponding survival rates of 90% and 81% over the same time periods [1].

With increasing numbers and improving survival rates, there is a growing population of long-term survivors following LT. This results in a shift of focus towards subjective patient outcomes, including quality of life (QoL), anxiety and depressive symptoms. Survival is easily quantifiable; patients’ subjective outcomes however are not. The last 20 years have seen the advent of a multitude of generic and disease-specific tools for measuring these patient-reported outcome measures (PROMs). Despite the increased recognition of the importance of PROMs and the growing number of tools, a standardized methodology for their application among patients undergoing LT has yet to be established.

The use of PROMs in the LT population is an invaluable tool to target improvements in clinical care, develop benchmarking standards and assess hospital performance [2]. Given the breadth of available tools (both generic and specific), it is difficult to select one that is most likely to deliver meaningful results and effect the most benefit in this cohort. Ultimately, the integration of a PROM into routine care of LT patients requires careful consideration at an early stage. Two systematic reviews by Jay et al. and Cleemput et al. reported on QoL instruments used in the LT population [3, 4]. However, both articles are over 10 years old and there have been significant methodological improvements since. Considering the above, a full, up to date systematic review is required. The aim of this systematic review is to provide a comprehensive overview of PROMs currently available for use in adults undergoing LT and their measurement properties.

## Methods

### Design

An initial scoping search was undertaken to identify relevant studies on this topic. This systematic review was conducted and written in compliance with the Preferred Reporting Items for Systematic Reviews and Meta-Analysis (PRISMA) guidelines and report in PROSPERO (PROSPERO registration number: CRD42021251533) [5].

### Search

A systematic search was conducted of MEDLINE, EMBASE, PubMed and COCHRANE to identify all studies including patients undergoing LT from January 2010 until August 2023. To report the screening process, the PRISMA flow diagram was used. Studies were included if they used a PROM to measure subjective insight of LT candidates and/or LT recipients, inclusive of QoL, anxiety, depressive symptoms, pain, mobility and liver failure symptoms. Included studies had to report either the development or evaluation of one or more measurement properties of their chosen PROM. Studies with non-original evaluations of the measurement properties were excluded. *In vitro* studies, studies only covering patients under 16 years of age or those reporting on living donors were excluded. Systematic literature reviews were excluded but were used to cross check included studies and identify additional references. Additionally, the reference list of included studies was reviewed to identify additional eligible studies. The complete search strategy is described in Supplementary Table S1.

### Screening Process

EndNote X7 (Clarivate Analytics, Pennsylvania, US) was used to collate the search results and exports of all citations were sent to the review software Rayyan (Qatar Computing Research Institute, Doha, Qatar) where duplicates were removed. After duplicate removal, four independent reviewers (SvK, SP, KJ, VW) screened by title and abstract and then by full text review. Abstracts that did not report enough information for an inclusion/exclusion decision underwent full text review. Disputes were resolved by the senior author (HH).

### Data Extraction

Data extraction elements were defined in advance and included: study population, demographics (age, sex, pre-/post-LT), the PROM tools (title, scoring system, number of items, domains) and measurement properties of the PROM. Some studies described measurement properties with different definitions. The COnsensus-based Standards for the selection of health Measurement Instruments (COSMIN) was used to ascertain which measurement properties were evaluated by the studies [6].

### Quality Assessment of Included Studies and Measurement Properties

Two authors (SvK, VW) first independently assessed the methodological quality of different domains of the studies using the COSMIN Risk of Bias checklist [7]. This employs a four-point rating system (“very good,” “adequate,” “doubtful” or “inadequate”) and the overall quality rating of each study is based on “the worst score counts” principle, i.e., the lowest rating of any standard. Table 1 presents information on the domains used to evaluate the risk of bias and quality of the measurement properties for each PROM.

**TABLE 1:** Description of the domains used to evaluate the risk of bias and quality of the measurement properties for each PROM.

Domain	Description
Reliability	
Internal consistency	The degree of the interrelatedness among the items of the PROM, as long as the items together form a unidimensional scale. Most of the times, the Cronbach’s alpha is measured. If the Cronbach’s alpha is >0.70, the internal consistency can be deemed “sufficient”
Reliability	The proportion of the total variance in the measurements which is due to “true” differences between patients. There must be evidence that the patients are stable at the time of the PROM assessment. If the intra class correlation coefficient is > 0.70, the reliability is deemed “sufficient”
Measurement error	The systematic and random error of a patient’s score that is not attributed to true changes in the construct to be measured. The smallest detectable change should be smaller than the minimal important change, to deem the measurement property “sufficient”
Validity	
Content validity	The degree to which the content of a PROM is an adequate reflection of the construct to be Measured. Content validity is considered the most important measurement property, because the items of the used PROM should be relevant, comprehensive and comprehensible for the patient population in which the PROM is used
Contruct validity	The degree to which the scores of a PROM are consistent with hypotheses based on the assumption that the PROM validly measures the construct to be measured Construct validity is divided into structural validity, hypotheses testing and cross-cultural validity *Structural validity* refers to the degree to which the scores of a PROM are an adequate reflection of the dimensionality of the construct to be measured and is usually assessed by factor analysis *Hypotheses testing for construct validity* refers to the degree to which the scores of a PROM are consistent with hypotheses *Cross‐cultural validity* refers to the degree to which the performance of the items on a translated or culturally adapted instrument are an adequate reflection of the performance of the items of the original version of the instrument. Therefore, this measurement has to be assessed by at least two different groups
Criterion validity	The degree to which the scores of a PROM are an adequate reflection of a ‘gold standard’, deemed ‘sufficient’ if the correlation with this gold standard is ≥0.70 or has an Area Under the Curve of ≥0.70
Responsiveness	The ability of a PROM to detect change over time in the construct to be measured. The results should be in accordance with the hypotheses or have an Area Under the Curve of ≥0.70
Interpretability	Interpretability is the degree to which one can assign qualitative meaning ‐ that is, clinical or commonly understood connotations – to a PROM’s quantitative scores or change in scores

### Data Synthesis

Subsequently, the quality of the measurement properties was assessed by the updated criteria for good measurement properties (based on Terwee et al, and Prinsen et al) as outlined by the COSMIN guideline for systematic reviews [6, 7].

Measurement properties were assessed using the following principles: content validity, structural validity, internal consistency, cross‐cultural validity, reliability, measurement error, criterion validity, hypothesis testing for construct validity and responsiveness. The quality of the measurement properties were scored using a four-point rating system (“+”= sufficient, “?” = indeterminate, “−“ = insufficients “±” = inconsistent). When the measurement properties of a PROM were not reported in any of the included articles, no score was assigned.

The criteria for good measurement properties were then applied to the results per measurement property per PROM, and the quality of the evidence (using the GRADE approach) was analyzed.

## Results

**FIGURE 1:**
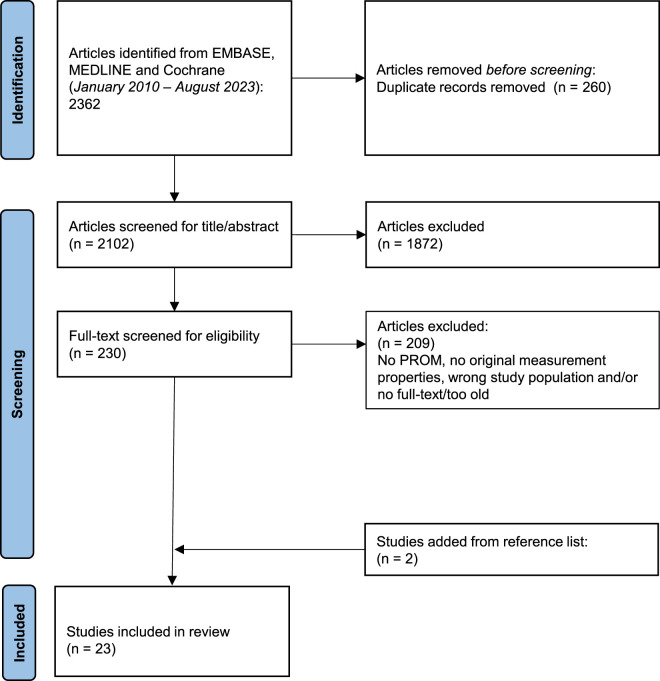
Flow diagram.

The search strategy retrieved a total of 2,362 titles/abstracts. After 260 duplicates were removed, 2,102 abstracts were screened, and 210 full-text articles were retrieved for further review. Following reference list and citation searching, two more articles were retrieved. After further review, a total number of 23 studies were included (Figure 1).

In total, 35 PROMs were used, with a minimum of one, and a maximum of six PROMs per study. PROMs could be divided in two categories: generic and disease-specific PROMs, and PROMs used for pre- and post-LT populations. Seven PROMs were disease-specific for liver disease and/or LT. Additionally, two PROMs addressed osteoporotic symptoms [Quality of Life Questionnaire in Osteoporosis (QUALIOST)] and emotional responses of organ transplant recipients [*the Transplant Effects Quesstionnaire (TxEQ)*], and were also categorized as disease-specific PROMs. 25 PROMs used in the studies were generic. One PROM was categorized under “utility measures,” providing utilities or values regarding health, that can be used for cost-utility analyses or interventions [8].

A total of eleven PROMs were applied to the pre-LT population, while thirteen were used for post-LT population. Additionally, eleven PROMs were used for both the pre- and post-LT population. Detailed study characteristics are described in Table 2, and a brief description of the PROMs evaluated is presented in Supplementary Table S2.

**TABLE 2:** Study and patient characteristics, categorized per Patient Reported Outcome Measurements (PROMs).

PROM	Author	Country	Publication year	Study population	Gender (male (%))	Age (mean (SD))	Mode of administration	Number of items	Response rate (%)	Target population	Patient population (pre-/post LT)
Disease-specific PROMs											
Short Form Liver Disease Quality of Life ((SF-)LDQOL)	Kanwal F (SF-LDQOL) [9]	USA	2008	156	54.8	53.9 (11)	Questionnaire	36			Pre
	Gralnek I.M [10].	USA	2000	221	64.3	52.2		111	86.6		Pre
Transplant Effects Questionnaire (TxEQ)	Pérez-San-Gregorio, MÁ [11]	Spain	2018	240				21			Post
	Annema, C [12].	Netherlands	2018	116	65.5	50.8 (11.4)	Questionnaire		75.8		Both
Post-Liver Transplant Quality of Life (pLTQ)	Molski, C [13].	Brazil	2016	160		56.9 (10.4)	Questionnaire	32			Post
	Saab, S [14].	USA	2011	196	59.7	53.1 (12.6)		32	93.8		Post
Self-made questionnaire	Parsa Yekta, Z [15].	Iran	2013	250	63.3	37.5 (12)	Questionnaire administered by hospital receptionist	40			Post
Self-made questionnaire	Lasker, J. N. (social QoL) [16]	USA	2011	100	0	58.5	Questionnaire via mail, online and interview		Response to items ranged from 93% to 100%	women with PBC on waiting list (WL) and post-transplant (PT)	Both
Self-made questionnaire	Franciosi, M. (ITaLi-Q) [17]	Italy	2011	177	71.8	57.2	Questionnaire, self-administered and anonymous	37	100% first questionairre, 49/177 the retest	Patients requiring HBV prophylaxis after LT	Post
Self-made questionnaire	Chen, X. (Post-LiverTransplant Symptom Experience Questionnaire) [18]	China	2021	265 (reliability tested on 30 patients in pilot study)	80		Questionnaire	40	96.1		Post
Self-management Questionnaire for LT recipients	Xing L [19].	China	2015	124				45			Post
Quality of Life Questionnaire in Osteopororis (QUALIOST)	Atamaz, F [20].	Turkey	2013	38 LT patients, 42 controls	81.6	42 (11.6)		24	ND		Post
Generic PROMs											
Short-form 36 (SF-36)	Fernandez, A. C [21].	USA	2016	125	60.8	56.1		36	96		Pre
	Miller-Matero, L. R [22].	USA	2014	84	66.8	SRD 53.96 (7.11) and HRD 55.87 (6.89)	Semi-structured interview	36	66.7		Both (prospective study)
Hospital Anxiety and Depression Score (HADS)	Pelgur H [23].	Turkey	2009	64	67		Face-to-face interview, Questionnaire administered by researcher	14	ND	patients who had undergone liver transplantation at least 1 month prior and were attending clinic for follow-up	Post
	Miller-Matero, L. R [22].	USA	2014	84	66.8	SRD 53.96 (7.11) and HRD 55.87 (6.89)	Semi-structured interview	14	66.7		Both (prospective study)
	Lin. X [24]	China	2017	285	75.8	53.3 (10.2)	Questionnaire	14	95		Post
World Health Organisation – Five Wellbeing Index (WHO-5)	Fernandez, A. C [21].	USA	2016	125	60.8	56.1 (8.64)	“	5	56		Pre
	Weber S [25].	Germany	2021	79	64.6	58.2	Questionnaire	5	ND		Post
WHOQOL-BREF	Annema, C [12].	Netherlands	2018	116	65.5	50.8 (11.4)	Questionnaire	24	75.8		Both
	Molski, C [13].	Brazil	2016	160		56.9 (10.4)	Questionnaire				Post
Post-Traumatic Growth Inventory (PTGI)	Gangeri, L [26].	Italy	2018	233	84	61	Questionnaire send to patients	21	76		Post
	Scrignaro M [30].	Italy	2016	100	15	59.88		21	58		Post
The Functional Assessment of Cancer Therapy - General (FACT-G)	Gangeri, L [26].	Italy	2018	233	84	61	Questionnaire send to patients	27	76	“	Post
Connor Davidson resilience scale (CD-RISC)	Fernandez, A. C [21].	USA	2016	125	60.8	56.1 (8.64)		25	56		Pre
Beck Depression Inventory (BDI)	Fernandez, A. C [21].	USA	2016	125	60.8	56.1 (8.64)		21	56		Pre
Beck Anxiety Inventory (BAI)	Fernandez, A. C [21].	USA	2016	125	60.8	56.1 (8.64)		21	56		Pre
Medical Outcomes Study Social Support Survey (SSS)	Fernandez, A. C [21].	USA	2016	125	60.8	56.1 (8.64)		20	56		Pre
State-Trait Anxiety Inventory (STAI-6)	Annema, C [12].	Netherlands	2018	116	65.5	50.8 (11.4)	Questionnaire	6	75.8		Both
Center of Epidemiological Studies Depression Scale (CES-D)	Annema, C [12].	Netherlands	2018	116	65.5	50.8 (11.4)	Questionnaire	20	75.8		Both
Pearlin-Scooler Mastery Scale	Annema, C [12].	Netherlands	2018	116	65.5	50.8 (11.4)	Questionnaire	7	75.8		Both
Coping Inventory for Stressful Situations (CISS-SF)	Annema, C [12].	Netherlands	2018	116	65.5	50.8 (11.4)	Questionnaire	21	75.8		Both
Perceived Social Support Scale (PSSS)	Lin, X [24].	China	2017	285	75.8	53.3 (10.2)	Questionnaire	14	95		Post
General Comfort Questionnaire	Demir B [29].	Turkey	2021	148	81.8%	ND	Interview	28	ND		Post
Fatigue Symptom Inventory (FSI)	Lin, X [24].	China	2017	285	75.8	53.3 (10.2)	Questionnaire	13	95		Post
Patient Health Questionnaire depression scale (PHQ-9)	Gronewold N [27].	Germany	2022	544	63.1	51.95 (9.84)	Questionnaire	9	ND		Pre
Generalized anxiety disorder screener (GAD-7)	Gronewold N [27].	Germany	2022	544	63.1	51.95 (9.84)	Questionnaire	7	ND		Pre
Perceived social support questionnaire	Gronewold N [27].	Germany	2022	544	63.1	51.95 (9.84)	Questionnaire	14	ND		Pre
Sense of Coherence Scale by Antonovsky	Gronewold N [27].	Germany	2022	544	63.1	51.95 (9.84)	Questionnaire	9	ND		Pre
General Self-Efficacy Short Scale	Gronewold N [27].	Germany	2022	544	63.1	51.95 (9.84)	Questionnaire	3	ND		Pre
German Body Image	Gronewold N [27].	Germany	2022	544	63.1	51.95 (9.84)	Questionnaire	20	ND		Pre
Short Questionnaire to Assess Health-Enhancing Physicial Activity (SQUASH)	Ushio M [28].	Japan	2023	173	47.4	ND	Questionnaire	13	ND		Post
UCLA Loneliness Scale	Weber S [25].	Germany	2021	79	64.6	58.2	Questionnaire	20	ND		Post
Utility Measure											
EQ-5D	Russell R.T [8].	USA	2009	285	64	53.3		5			Both

Abbreviation: ND = not described.

The risk of bias and methodological qualities of the PROMs used and described in the selected studies are described in Tables 3, 4, respectively. Overall, the evidence for the measurement properties was limited and the methodological quality was insufficient or inconsistent. None of the studies evaluated all measurement properties of the COSMIN system. Internal consistency was the most evaluated measurement property.

**TABLE 3:** Risk of Bias using the COnsensus-based Standards for the selection of health Measurement Instruments (COSMIN) Risk of Bias checklist.

PROM	Author	Content valicity	Structural validity	Internal valdity (Cronbach’s alpha)	Cross-cultural validity	Reliability	Measurement error (test-retest)	Criterion validity	Hypothesis testing for construct validity	Responsiveness
Disease specific N = 9										
(SF-)LDQOL	Kanwal F (SF-LDQOL)		Inadequate	very good			adequate	very good	very good	very good
	Gralnek I.M.	Very good	Inadequate	very good			inadequate		very good	
TxEQ	Pérez-San-Gregorio, MÁ			very good	very good					
	Annema, C		inadequate	very good	inadequate					
pLTQ	Molski, C			very good	very good	very good				very good
	Saab, S		very good	very good			very good	very good		
Self-made questionnaire	Parsa Yekta, Z	very good	Inadequate	very good		adequate				
Self-made questionnaire	Lasker, J. N. (social QoL)			very good		inadequate	inadequate		doubtful	
Self-made questionnaire	Franciosi, M. (ITaLi-Q)			very good		doubtful	very good	very good	very good	
Self-made questionnaire	Chen, X. (Post-LiverTransplant Symptom Experience Questionnaire)		Inadequate	Very good					inadequate	
Self-management Questionnaire for LT recipients	Xing L			very good						
QUALIOST	Atamaz, F		NA	Very good	Doubtful	doubtful		Very good		
Generic N = 26										
Short-form 36 (SF-36)	Fernandez, A. C			Very good		Inadequate			Very good	
	Miller-Matero, L. R			very good		very good			very good	
Hospital Anxiety and Depression Score (HADS)	Pelgur H			very good						
	Miller-Matero, L. R			very good		very good			very good	
	Lin. X			Very good						
World Health Organisation – Five Wellbeing Index (WHO-5)	Fernandez, A. C			Inadequate/Doubtful						
	Weber S			Doubtful						
WHOQOL-BREF	Annema, C			very good	inadequate					
	Molski, C									
Post-Traumatic Growth Inventory (PTGI)	Gangeri, L			very good	doubtful		very good		Doubtful	very good
	Scrignaro M			Very good		Inadequate	Inadequate		Very good	
The Functional Assessment of Cancer Therapy - General (FACT-G)	Gangeri, L			very good	doubtful		very good		Doubtful	very good
Connor Davidson resilience scale (CD-RISC)	Fernandez, A. C		inadequate	very good		adequate			very good	
Beck Depression Inventory (BDI)	Fernandez, A. C			Inadequate/Doubtful						
Beck Anxiety Inventory (BAI)	Fernandez, A. C			Inadequate/Doubtful						
Medical Outcomes Study Social Support Survey (SSS)	Fernandez, A. C			Inadequate/Doubtful						
State-Trait Anxiety Inventory (STAI-6)	Annema, C			Inadequate/Doubtful						
Center of Epidemiological Studies Depression Scale (CES-D)	Annema, C			Inadequate/Doubtful						
Pearlin-Scooler Mastery Scale	Annema, C			Inadequate/Doubtful						
Coping Inventory for Stressful Situations (CISS-SF)	Annema, C			very good						
Perceived Social Support Scale (PSSS)	Lin. X			Very good						
General Comfort Questionnaire	Demir B			Doubtful	Inadequate					
Fatigue Symptom Inventory (FSI)	Lin. X			Very good						
Patient Health Questionnaire depression scale (PHQ-9)	Gronewold N			Doubtful						
Generalized anxiety deisorder screener (GAD-7)	Gronewold N			Doubtful						
Perceived social support questionnaire	Gronewold N			Doubtful						
Sense of coherence scale by Antonovsky	Gronewold N			Doubtful						
general self-efficacy short scale	Gronewold N			Doubtful						
German body image	Gronewold N			Very good						
Short Questionnaire to Assess Health-Enhancing Physicial Activity (SQUASH)	Ushio M					Adequate	adequate	Very good		
UCLA loniless scale	Weber S			Doubtful						
Utility measures N = 1										
EQ-5D	Russell R.T.					Doubtful	inadequate	very good	very good	

**TABLE 4:** Quality Assessment of the Patient Reported Outcome Measures (PROMs) using the COnsensus-based Standards for the selection of health Measurement Instruments (COSMIN) guideline.

PROM	Author	Content Validity	Structural validity	Internal valdity	Cross-cultural validity	Reliability	Measurement error	Criterion validity	Hypothesis testing for construct validity	Responsiveness
Disease specific N = 9										
(SF-)LDQOL	Kanwal F (SF-LDQOL)		?	-				?	+	+
	Gralnek I.M.		?	-					+	
TxEQ	Pérez-San-Gregorio, MÁ			+	+					
	Annema, C.			-	?					
pLTQ	Molski, C.			+	?	+				+
	Saab, S.		?	+			-	?		
Self-made questionnaire	Parsa Yekta, Z.		?	+		?				
Self-made questionnaire	Lasker, J. N. (social QoL)		?	-		?	?		?	
Self-made questionnaire	Franciosi, M. (ITaLi-Q)		?/-	+		?		+	?	
Self-made questionnaire	Chen, X. (Post-LiverTransplant Symptom Experience Questionnaire)		?	+						
Self-management Questionnaire for LT recipients	Xing L.			+						
QUALIOST	Atamaz, F.			+	+	+		-		
Generic N = 26										
Short-form 36 (SF-36)	Fernandez, A. C.			+		?			+	
	Miller-Matero, L. R.			+					+	
Hospital Anxiety and Depression Score (HADS)	Pelgur H.			+						
	Miller-Matero, L. R.			+					+	
	Lin. X			+						
World Health Organisation – Five Wellbeing Index (WHO-5)	Fernandez, A. C			+						
	Weber S.			+						
WHOQOL-BREF	Annema, C.			-						
	Molski, C.									
Post-Traumatic Growth Inventory (PTGI)	Gangeri, L.			+			?		?	+
	Scrignaro M.			+		?	?		+	
The Functional Assessment of Cancer Therapy - General (FACT-G)	Gangeri, L.			+			?		?	+
Connor Davidson resilience scale (CD-RISC)	Fernandez, A. C.			+					?	
Beck Depression Inventory (BDI)	Fernandez, A. C.			+						
Beck Anxiety Inventory (BAI)	Fernandez, A. C.			+						
Medical Outcomes Study Social Support Survey (SSS)	Fernandez, A. C.			+						
State-Trait Anxiety Inventory (STAI-6)	Annema, C.			+						
Center of Epidemiological Studies Depression Scale (CES-D)	Annema, C.			+						
Pearlin-Scooler Mastery Scale	Annema, C			+						
Coping Inventory for Stressful Situations (CISS-SF)	Annema, C			+						
Perceived Social Support Scale (PSSS)	Lin. X			+						
General Comfort Questionnaire	Demir B			+	?					
Fatigue Symptom Inventory (FSI)	Lin. X			+						
Patient Health Questionnaire depression scale (PHQ-9)	Gronewold N			+						
Generalized anxiety disorder screener (GAD-7)	Gronewold N			+						
Perceived social support questionnaire	Gronewold N			+						
Sense of coherence scale by Antonovsky	Gronewold N			+						
General self-efficacy short scale	Gronewold N			+						
German body image	Gronewold N			+						
Short Questionnaire to Assess Health-Enhancing Physicial Activity (SQUASH)	Ushio M					-	?	?		
UCLA loniless scale	Weber S			+						
Utility measures N = 1										
EQ-5D	Russell R.T.						?	-	+	

Abbreviations: + = positive rating; ? = indeterminate rating; − = negative rating.

### Disease-Specific PROMs

A total of twelve articles described the measurement properties of the nine disease-specific PROMs [9–20]. Of these PROMs, one was used in a pre-LT population, six in the post-LT population and two in both the pre- and post-LT population.

Only the (*Short-form) Liver Disease Quality of Life* [(SF-)LDQOL] (two studies), *TxEQ* (two studies) and *Post-Liver Transplant Quality of Life* (pLTQ) (two studies) were employed by multiple studies, each with their own measurement properties of the utilized PROMs. The *pLTQ* scored a high evidence level for internal validity, reliability and responsiveness.

The *ITaLi-Q*, the *self-made questionnaires* by Parsa Yekta et al. and Chen et al., the *self-management questionnaire for LT-recipients* by Xing et al. and the *QUALIOST* were all graded with a high evidence level for adequate internal validity [15, 18, 19].

The *QUALIOST* reported a high level of evidence for cross-cultural validity and reliability. The *(SF-)LDQOL* reported a high level of evidence on hypothesis testing for construct validity and responsiveness.

### Generic PROMs

A total of fourteen articles described the measurement properties of 26 generic PROMs [12, 13, 21–30]. Of these PROMs, ten were used in a pre-LT population, and eight in the post-LT population. Furthermore, eight PROMs were utilized in both the pre- and post-LT population. The *EQ-5D*, graded as a ‘utility measure’, used in both pre- and post-LT population.

The most utilized PROMs were the *Hospital Anxiety and Depression Score* (HADS) (three studies), the *Short-form 36* (SF-36) (two studies), the *World Health Organisation – Five Wellbeing index* (WHO-5) (two studies), the *WHOQOL-BREF* (two studies) and the *Post-Traumatic Growth Inventory* (PTGI). All other PROMs were used by one study only.

There was moderate evidence for the internal validity in most studies; the *HADS* and *SF-36* both scored a high level of evidence in internal validity and hypothesis testing for construct validity. The *Short-Questionnaire to Assess Health-Enhancing Physical Activity* showed a low level of evidence for reliability. The *EQ-5D* showed a low level of evidence for criterion validity.

## Discussion

This systematic review is the first study to evaluate the methodological quality of PROMs utilized in the pre- and post-LT population, using the COSMIN-guidelines. In total, 23 articles employed nine disease-specific PROMs for the pre- and post-LT population, while 25 general PROMs and one utility measure were included. The (SF-)LDQOL, TxEQ and pLTQ were the most commonly used disease-specific PROMs. PLTQ showed high quality evidence of Internal validity, reliability and responsiveness. HADS was the most frequently used general PROM, and showed high-quality evidence for internal consistency and hypothesis testing for construct validity.

The methodological quality of most general and disease-specific PROMs was found to be limited, as the majority of the studies failed to adequately evaluate the measurement properties of the utilized PROMs, a trend observed in other similar reviews [31–33]. Within this review, most studies merely described the internal validity, while other essential measurement properties either lacked a description or exhibited inadequate methodological quality. Furthermore, there was inconsistency in scores for different measurement properties between different studies. For example, internal validity of the PROM *TxEQ* demonstrated sufficient quality in one study, but insufficient quality in another study, while both studies utilized the same PROM within the post-LT patient population. This discrepancy aligns with finding from the study by Elberts et al., who evaluated the quality of measurement properties in patients with neurological diseases [32]. Variations in measurement properties between studies can be in part attributed to differences in patient demographics and socio-economic characteristics. McHorney et al. found that SF-36 scores were generally lower among the elderly, those with less than a high school education and those in poverty [34]. Therefore, socio-economic backgrounds and diverse patient populations must be considered when implementing a PROM.

The limited use of PROMs in this patient population made it challenging to effectively synthesize and summarize the data. Most PROMs were reported in only one study, with only thirteen studies evaluating the same PROMs [9, 10, 14]. This lack of quality assessment is also reflected in reviews evaluating PROMs in other medical subpopulations [32, 33]. Aiyegbus et al. reviewed the measurement properties of PROMs used in kidney transplantation patients [31]. Despite a greater quantity of studies including a quality assessment of PROMs, the evidence was still of poor quality, with significant gaps in information. Chiarotto et al. evaluated the quality of measurement properties in PROMs for patients with lower back pain – including the *SF-36, SF-12, EQ-5D-3L, EQ-5D-5L, Nottingham Health Profile* and the *PROMIS-GH-10*, and found similar scarcities of high-quality evidence in their patient population [35].

The lack of robust quality assessment of PROMs can be attributed to their relatively recent rise in prominence in clinical research. However, PROMs are of the upmost importance for individual patients, as they reflect what matters to patients at a personal level, transcending the broader context of population-level survival. Therefore, identifying high quality, high level of evidence measures that can be standardized across patient populations is of paramount importance.

Assessing subjective patient measurements remain complex due to variability in individual values. Individuals prioritize different aspects of their live, posing a challenge in developing a universally applicable tool. While general tools like the SF-36 and HADS offer a broad applicability, they lack assessment of disease-specific burden. Disease-specific PROMs are therefore more suitable for subpopulations, facilitating accurate detection of burden in subjective measurements.

An additional consideration when selecting a PROM is its original intended purpose. For example, the EuroQol-5 Dimension (EQ-5D) was not originally conceived for the evaluation of QoL in medical research but rather to facilitate cost-effectiveness assessments, rendering it particularly valuable in economic studies. Poor definitions within PROMs also pose a problem, for example, the definition of HRQoL is not always clear [36].

This review extends beyond PROMs simply assessing QoL, to encompass an overview of all PROMs used in pre- and post-LT population. There is not a clear single best option and the choice of a PROM should be made with careful deliberation, considering the particular objectives of the study. Over the last decade, the use of PROMs has increased, including the use of web questionnaires [37]. The integration of PROMs into research and clinical practice enables more accurate assessment of patient symptoms and supports more efficient allocation of healthcare resources. In the context of LT, evaluating changes in symptoms before and after the procedure is particularly relevant, as it could reflects treatment effectiveness. Disease-specific PROMs are therefore generally more appropriate for assessing disease-related symptoms with greater sensitivity. In contrast, generic PROMs are more appropriate to compare across different diseases and populations, and preferred in health technology assessment [38]. Nonetheless, the use of both generic and disease-specific PROMs requires careful consideration. When clinicians or researchers select existing PROMs or developing new ones, several critical aspects must be addressed, including cross-cultural validation, the intended purpose (clinical or research), and patient acceptability and feasibility [31].

There are limitations to this review. Firstly, the populations of the included studies are heterogenous, conducted across many different countries and languages. Cultural nuances play a pivotal role in shaping perception, and the translation of PROMs into different languages may introduce variations in interpretation. Cross-cultural validation represents one approach addressing this problem. However, most of the studies did not provide a comprehensive report on this measurement property. Furthermore, the pre- and post-LT populations have different considerations, including underlying liver disease, the severity of the disease, time after transplantation and the current symptoms of the patient. All these aspects influence patient’s subjective feelings and therefore the outcome of the PROM utilized. However, since there was a lack of strong evidence studies, these sub-analyses could not be performed.

In summary, this review identified the *(SF-)LDQOL, TxEQ and pLTQ* as the most commonly used disease-specific PROMs, and the *HADS* was the most frequently used general PROM. For disease-specific PROMs in both pre- and post-LT patients, the *pLTQ* emerges as the PROM of choice based on its superior methodological quality. However, the limited number of studies assessing the quality of the same PROMs and the low quality of evidence surrounding these instruments highlight the necessity of further investigation. Further studies are needed to carefully evaluate both the appropriateness of the PROM selection for their target population, and the evidence regarding the measurement properties of these instruments, either through rigorous assessment or validation.

## Data Availability

The original contributions presented in the study are included in the article/Supplementary Material, further inquiries can be directed to the corresponding author.

## Abbreviations

BAI, Beck Anxiety Inventory; BDI, Beck Depression Inventory; CD-RISK, Connor Davidson Resilience Scale; CES-D, Center of Epidemiological Studies Depression Scale; CISS-SF, Coping Inventory for Stressful Situations; COSMIN, COnsensus-based Standards for the selection of health Measurement Instruments; EQ-5D, EuroQol-5 Dimension; FACT-G, The Functional Assessment of Cancer Therapy – General; FSI, Fatigue Symptom Inventory; GAD-7, Generalized anxiety disorder screener; HADS, Hospital Anxiety and Depression Score LPA-SQUASH: Light-intensity Physical Activity Short Questionnaire to Assess Health-Enhancing Physicial Activity LT, Liver Transplantation PHQ-9, Patient Health Questionnaire depression scale pLTQ, Post-Liver Transplant Quality of Life PRISMA, Preferred Reporting Items for Systematic Reviews and Meta-Analysis PROM, Patient Reported Outcome Measures PSSS, Perceived Social Support Scale PTGI, Post-Traumatic Growth Inventory QoL, Quality of Life; QUALIOST, Quality of Life Questionnaire in Osteopororis (SF-)LDQoL, Short Form Liver Disease Quality of Life SF-36, Short-Form 36 SOC-L9, Sense of Coherence scale by Antonovsky SSS, Medical Outcomes Study Social Support Survey STAI-6, State-Trait Anxiety Inventory TxEQ, Transplant Effects Questionnaire WHO-5, World Health Organisation – Five Wellbeing Index.
