# Supplementary material for: Quality of Measurement Properties in Patient Reported Outcomes Used in Adult Liver Transplant Candidates and Recipients: a Systematic Review
Source: Transpl Int. 2025 Oct 2;38:14497. doi: 10.3389/ti.2025.14497 (PMC12529315; doi:10.3389/ti.2025.14497)
Supplement: Supplementary file 1 [file DataSheet1.pdf]

Supplementary material

Quality of Measurement Properties in Patient Reported Outcomes used in Adult Liver Transplant Candidates and Recipients: A Systematic Review

Samira E.M. van Knippenberg M.D.[1]*, Sarah F. Powell-Brett MBChB., Ph.D [2,3]*, Kunal Joshi M.D.[2], Víola B. Weeda M.D. Ph.D.[4], Hermien Hartog M.D., Ph.D.[3,5]

1. Department of Surgery, Amsterdam University Medical Centers, Amsterdam, The Netherlands
2. The Liver Unit, Queen Elizabeth Hospital Birmingham, Birmingham, United Kingdom
3. Centre for Liver and Gastrointestinal Research, Institute of Immunology and Immunotherapy, University of Birmingham, Birmingham, United Kingdom
4. Department of Surgery, Hôpital Universitaire de Bruxelles, Bruxelles, Belgium
5. Department of Surgery, Section of HPB & Liver Transplantation, University Medical Center Groningen, Groningen, the Netherlands

*Both authors contributed equally

Corresponding author: H. Hartog, h.hartog@umcg.nl
Department of Surgery
UMC Groningen, 9713 GZ Groningen, The Netherlands
P: +31 50 361 28 96

Supplementary Table 1. Search strategy

**Patient Reported Outcome Measures (PROMs)**	**Liver transplant**
"HRQL"[Title/Abstract] OR "HRQOL"[Title/Abstract] OR "QOL"[Title/Abstract] OR<br><br>"Quality of life"[Title/Abstract] OR<br><br>"health index*"[Title/Abstract] OR<br><br>"health indices"[Title/Abstract] OR<br><br>"health profile*"[Title/Abstract] OR<br><br>"health status"[Title/Abstract] OR "PROM"[Title/Abstract] OR "PRO"[Title/Abstract] OR "PROMS" OR<br><br>"patient reported outcome*"[Title/Abstract] OR "self assessed outcome*"[Title/Abstract] OR "patient assessed outcome*"[Title/Abstract] OR "self report outcome*"[Title/Abstract] OR "health utility"[Title/Abstract] OR<br><br>"patient report outcome*"[Title/Abstract] OR "patient report measure*"[Title/Abstract] OR "self report measure*"[Title/Abstract] OR	"liver transplant*"[Title] OR "hepatic transplant*"[Title] OR "liver allograft*"[Title] OR<br><br>"hepatic allograft*"[Title] OR<br><br>"liver allotransplant*"[Title] OR "hepatic allotransplant*"[Title] OR "liver transplantation"[MeSH Terms]

"self report assessment*"[Title/Abstract] OR "self assess*"[Title/Abstract]<br><br>'Patient reported outcome measure'[MeSH Terms])	
1 AND 2	Filtered for last 10 years<br><br>English language available

Supplementary Table I. Search strategy; Search terms included controlled terms from Mesh in PubMed and Emtree in Embase as well as free text terms.

Supplementary Table 2: Description of the Patient Reported Outcome Measures (PROMs)

Patient Reported Outcome Measures (PROM)	Description
**Measuring health that is specific to a particular disease, set of conditions or part of the body**	
**Short Form Liver Disease Quality of Life ((SF-)LDQoL)** [12, 13]	The LDQOL is a disease-targeted, self-completed, multidimensional HRQOL instrument for patients with advanced chronic liver disease. The questionnaire containing the SF-36 Health Survey version 2.0 as the generic core, supplemented by disease-targeted scales. The disease-targeted supplement of the LDQOL 1.0 includes 12 additional multi-item scales, as follows: liver disease–related symptoms (17 items), liver disease–related effects on activities of daily living (10 items), concentration (seven items), memory (six items), sexual functioning (three items), sexual problems (three items), sleep (five items), loneliness (five items), hopelessness (four items), quality of social interaction (five items), health distress (four items), and self-perceived stigma of liver disease (six items).<br><br>The Short Form LDQOL includes 36 disease-targeted items representing nine domains: symptoms of liver disease, effects of liver disease, memory⁄concentration, sleep, hopelessness, distress, loneliness, stigma of liver disease and sexual problems. Items within scales are averaged together and scale scores are linearly transformed to a 0–100 possible range, where higher scores denote better HRQOL.

**Transplant Effects Questionnaire (TxEQ)** [42]	Measures the emotional response to the receipt of a transplanted organ using 21 items with three subscales: worries about the transplant, feelings of guilt toward the donor, and disclosure about having had a transplant. Items are scored on a five-point Likert scale (1 = "strongly disagree"; 5 = "strongly agree"). On the subscales "worry" and "guilt," a higher score indicates a problematic response, whereas on the "disclosure" subscale, a lower score indicates a problematic response.
**Post-Liver Transplant Quality of Life (pLTQ)** [17]	The survey assesses specific factors that can affect the lives of patients who have undergone liver transplantation, including symptoms, mood, limitation of the activities of daily living, energy level, and transplant-related care. It comprises 32 items, grouped into eight domains: Emotional (four items), Worry (seven items), Medications (four items), Physical Function (six items), Healthcare (four items), Graft Rejection Concern (two items), Financial (two items), and Pain (three items). Items are scored on a seven-point Likert-type scale, and 1 corresponds to "always" and 7 to "never".
**Self-made questionnaire** [18]	The questionnaire consists of three factors (health satisfaction, concerns and complications) and 40 items with a four-point Likert scale assessing the QoL in liver transplant recipients.
**Self-made questionnaire** [19]	The questionnaire was made out of multiple existing questionnaires and measures (e.g. fatigue, depression, coping, uncertainty, social support, stigma and QoL), as well as new measures with no appropriate standardized measures (e.g. liver disease symptoms, medication effects, fear/anxiety, information and social QoL).

**ITaLi-Q** [20]	Tool for measuring the impact of hepatitis B immune globulin on HRQOL and treatment satisfaction among LT recipients. It includes 41 items and covers 5 domains (side effects, positive and negative feelings, impact on the flexibility of daily activities, support, and satisfaction). [2019181717171717171711]
**Post-Liver Transplant Symptom Experience Questionnaire** [21]	Post-Liver Transplant Symptom Experience Questionnaire. It contained 40 items; each item represented one symptom and included three dimensions: frequency, intensity, and distress. If the participant reported the experience of a symptom within the past week, he or she was asked to rate its frequency, severity, and distress. Symptom frequency was rated on a numeric rating scale (NRS) from 0 (none) to 4 (ever-present). [2120191818181818181812]
**Self-management Questionnaire for LT-recipients, Translation of a Chinese chronic disease self-management program questionnaire code book** [22]	Includes two major parts: self-management and self-efficacy. Self-management included four sub-categories: exercise, cognitive symptom management, communication with physicians, and lifestyle management, with a total of 39 items. The subgroup self-efficacy included 6 items.
**Quality of Life Questionnaire in Osteopororis (QUALIOST)** [43]	This tool comprises 23 items designed to measure the QoL over the previous 4 weeks in osteoporotic patients and was developed to be used in conjunction with Short Form-36 (SF-36).
**Generic measures: measuring health in a general manner, can be used for various health conditions**	

**Short-form 36 (SF-36)** [44]	A 36-item HRQOL measure with 8 scales: physical functioning, [18 17 16 15 15 15 15 15 15] physical role, bodily pain, general health, vitality, social functioning, emotional role, and mental health. An additional 1-item measure of self-evaluated change in health status is available. The Likert rating method is used and raw scores are linearly transformed into 0 to 100 scales with higher transformed scores indicating better HRQOL.
**Hospital Anxiety and Depression Score (HADS)** [45]	A tool devised to measure anxiety and depression in a general medical population of patients. Both the anxiety and depression subscales contain 7 items with a 4 point Likert scale, which contains questions regarding the past 4 weeks. A score of 7 or less is considered normal.
**World Health Organisation – Five Well-Being Index (WHO-5)** [46]	This tool is a short self-reported measure of current mental wellbeing with 5 items. The five items are: (1) “I have felt cheerful in good spirits”, (2) “I have felt calm and relaxed”, (3) “I have felt active and vigorous”, (4) “I woke up feeling fresh and rested”, (5) “My daily life has been filled with things that interest me”. statement can be answered from ‘all of the time’ till ‘at no time’. The raw score is calculated by totaling the score representing each answer and ranges from 0 to 25. [2423222121212121212115]
**WHOQOL-BREF** [47]	This is a short versions of the WHOQoL-100. It includes 26 items, of which 24 are devided in four domains, and the remaining 2 items regard the general QoL. A higher domain score equals a higher QoL.
**Post-Traumatic Growth Inventory (PTGI)** [48]	The tool assesses post-trauma growth an self-improvement. It consists of 21 items answered on a 6-point scale, and scores on 5 domains of life: 1. Relationship with others, 2: new possibilities, appreciation of life, spiritual change, personal strength.

**The Functional Assessment of Cancer Therapy - General (FACT-G)** [49]	Is measures four domains of health related quality of life in cancer patients. The questionnaire includes 27 items with a 5 point Likert scale ranging from 0 ("not at all") to 4 ("very much"). The scale in four domains: Physical, social, emotional and functional well-being.
**Connor Davidson Resilience Scale (CD-RISK)** [50]	This measures psychological resilience including 5 factors: 1. assesses personal competence, high standards and tenacity, 2. trust in one's instincts, tolerance of negative affect and strengthen effects of stress, 3. positive acceptance of change and secure relationships, 4 control, 5. Spirituality.
**Beck Depression Inventory (BDI)** [51]	The BDI is a widely used psychometric test for assessing the severity of depression. It contains 21-items with a 4 point Likert scale. Higher scores indicate more depressive symptoms.
**Beck Anxiety Inventory (BAI)** [52]	This tool has 21-items with a 4 point Likert scale that assesses symptoms of anxiety. It has a total score of 63. The higher the scores indicate more severy anxiety symptoms.
**Medical Outcomes Study Social Support Survey (SSS)** [53]	The SSS assesses the percieved social support in 20 items, in a 5 point Likert scale. For each item, patients are asked to indicate how often each kind of support was available to them if they needed it. Scores ranged from 0 (None of the time) to 4 (All of the time). Higher scores on the SSS reflect higher levels of perceived social support.
**State-Trait Anxiety Inventory**	The STAI-6 consists of six items rated on a four-point intensity scale (1 = "not at all"; 4 = "very much"), resulting in a total sum score between 6 and 24. Higher scores indicate more symptoms of anxiety. Based on a transformation of the original

**(STAI-6)** [54]	cutoff of 40 or greater for the general population found in the 20-item scale, a cutoff score of 12 or greater is used to identify clinically relevant cases.
**Center of Epidemiological Studies Depression Scale (CES-D)** [55]	This scale assesses symptoms of depression. It consists of 20 items rated on a four point likert scale (0 = "seldom or never"; 4 = "most of the time or always"). Higher scores indicate more symptoms of depression. A cutoff score of 16 or greater is used to identify clinically relevant cases.
**Pearlin-Scooler Mastery Scale** [56]	The mastery scale measures the degree to which individuals feel they can control things that happen to them and consists of seven items rated on a five-point Likert scale (1 = "totally disagree"; 5 = "totally agree"). Higher scores indicate a higher level of personal control.
**Coping Inventory for Stressful Situations (CISS-SF)** [57]	It has 21 items rated on a 5 point Likert scale to measure one's coping style in three dimensions of coping: task-oriented coping, emotion-oriented coping, avoidance coping. On the subscales "worry" and "guilt," a higher score indicates a problematic response, whereas on the "disclosure" subscale, a lower score indicates a problematic response.
**Perceived Social Support Scale (PSSS)** [58]	A short instrument designed to measure an individual's perception of support from 3 sources: family, friends and a significant other. This instrument is 12 questions long and the items are divided into three subscales relating to the source of the support (family, friends and significant other). Each of these subscales consists of four items, and each item ranges from very strongly disagree (score=1) to very strongly agree (score=7).

**Fatigue Symptom Inventory (FSI)** [59]	A 13-item instrument measuring the fatigue intensity (4 items), duration of fatigue (2 items) and the extent to which fatigue interferes with the quality of life (7 items) with a 11 point Likert scale. A higher score indicates a more intense fatigue experiences by the patient.
**Patient Health Questionnaire Depression scale (PHQ-9)** [60]	The instrument contains 9 items with a 4 point Likert scale, measuring depressive symptoms. Higher scores indicate more depressive symptoms. Cutoff point include: no depression (<5), mild depression (5–9), moderate (10–14), moderately severe (15–19), and severe (20–27).
**Generalized anxiety disorder screener (GAD-7)** [61]	A 7-item instrument for measuring ones anxiety during the last two weeks. Each items has a 4 point Likert scale.
**Short form Perceived social support questionnaire** [62]	The instrument includes 14 items, measuring the perceived social support on a 5 point Likert scale. Higher scores indicate a more perceived support.
**General Comfort Questionnaire** [63]	An instrument indicating patient comfort. The instrument contains 28 items with three subscales: relief (9 items), ease (9 items) and transcendence (10 items).

**Sense of coherence scale by Antonovsky (SOC-L9)** [64]	A 9-item instrument with 7 point Likert scale, measuring ones sense of coherence.
**General Self-efficacy Short Scale** [65]	The instrument consists of three items rated on a 5 point Likert scale, measuring the ones perception of disease, locus of control of reinforcement and self-efficacy
**German body image** [66]	This instrument measures the negative association with the own body of the patient, and vital body dynamics, refering to the perception of the own body regarding movement and physical activity. The instrument includes 10 items on a 5 point Liker scale, with higher scores indicating a more rejecting body evaluation.
**Light-intensity Physical Activity Short Questionnaire to Assess Health-Enhancing Physicial Activity (LPA-SQUASH)** [31]	The instrument is a modified version of the SQUASH-instrument [67], containing 13 items that show typical physical activities for four activity domains: transport, leisure, housework, and work. The items are described with specific physical activities and one's postures during these activities, such as sitting or standing. The instrument has a 4 point Likert scale ("not relevant", "somewhat relevant", "quite relevant", "highly relevant").

**UCLA loneliness scale** [68]	The instrument measures the feeling of loneliness and social isolation. It contains 20 items with a 5 point Likert scale ("not at all" to "absolutely"). After analysis, the overall score of the instrument ranges from 1 (lowest level of loneliness) to 5 (highest level to loneliness).
**Utility measures: These provide utilities or values regarding health and can be used for cost-utility analyses of interventions**	
**EuroQol-5 Dimension (EQ-5D)** [69]	A utility measure with a self-classifier and a visual analogue scale (VAS) which can be used to value health states. The self-classifier includes 5 dimensions: (i) mobility [18] self-care (iii) usual activities (iv) pain/discomfort (v) anxiety/depression. Each dimension has 3 levels of severity (no problems, some problems, and severe problems) and it is possible to describe 243 health states between 0 (dead) and 1 (perfect health). [11]
